# Lexical Specification of Prosodic Information in Swedish: Evidence from Mismatch Negativity

**DOI:** 10.3389/fnins.2016.00533

**Published:** 2016-11-29

**Authors:** Hatice Zora, Tomas Riad, Iris-Corinna Schwarz, Mattias Heldner

**Affiliations:** ^1^Department of Linguistics, Stockholm UniversityStockholm, Sweden; ^2^Department of Swedish Language and Multilingualism, Stockholm UniversityStockholm, Sweden

**Keywords:** Swedish stress, morphology, prosody, memory trace, EEG, MMN

## Abstract

Like that of many other Germanic languages, the stress system of Swedish has mainly undergone phonological analysis. Recently, however, researchers have begun to recognize the central role of morphology in these systems. Similar to the lexical specification of tonal accent, the Swedish stress system is claimed to be morphologically determined and morphemes are thus categorized as prosodically specified and prosodically unspecified. Prosodically specified morphemes bear stress information as part of their lexical representations and are classified as tonic (i.e., lexically stressed), pretonic and posttonic, whereas prosodically unspecified morphemes receive stress through a phonological rule that is right-edge oriented, but is sensitive to prosodic specification at that edge. The presence of prosodic specification is inferred from vowel quality and vowel quantity; if stress moves elsewhere, vowel quality and quantity change radically in phonologically stressed morphemes, whereas traces of stress remain in lexically stressed morphemes. The present study is the first to investigate whether stress is a lexical property of Swedish morphemes by comparing mismatch negativity (MMN) responses to vowel quality and quantity changes in phonologically stressed and lexically stressed words. In a passive oddball paradigm, 15 native speakers of Swedish were presented with standards and deviants, which differed from the standards in formant frequency and duration. Given that vowel quality and quantity changes are associated with morphological derivations only in phonologically stressed words, MMN responses are expected to be greater in phonologically stressed words than in lexically stressed words that lack such an association. The results indicated that the processing differences between phonologically and lexically stressed words were reflected in the amplitude and topography of MMN responses. Confirming the expectation, MMN amplitude was greater for the phonologically stressed word than for the lexically stressed word and showed a more widespread topographic distribution. The brain did not only detect vowel quality and quantity changes but also used them to activate memory traces associated with derivations. The present study therefore implies that morphology is directly involved in the Swedish stress system and that changes in phonological shape due to stress shift cue upcoming stress and potential addition of a morpheme.

## Introduction

Unlike most Germanic languages, Swedish exhibits both a stress system and a tone accent system^1^. While tonal accents have been investigated extensively, stress has received relatively little attention in Swedish. Stress is indeed more fundamental than accent in Swedish for the following reasons (Zonneveld et al., 1999): (i) As part of the rhythmical structure of the language, stress determines the phonetic quality of sounds; (ii) unlike accents, stress is a common feature of all dialects; and (iii) accents are not independent from stress. The present study, therefore, investigates Swedish stress, specifically the interplay between the stress system and morphology.

Like that of many other Germanic languages, the stress system of Swedish has mostly undergone phonological analysis. Stress systems of Germanic languages have typically been investigated based on phonological information such as word edge and number of syllables (Linell, 1972; van der Hulst, 1984; Kager, 1989; Wiese, 1996; Zonneveld et al., 1999; Kristoffersen, 2000; Shokri, 2001; Frid, 2003). Exceptions have been treated with features such as extrametricality^2^ and direct marking for exceptional behavior, whereas morphological information has not received much attention. Recently, however, researchers have begun to recognize the central role that morphology plays in stress systems.

Riad (2012, 2014) argues that stress placement in Swedish largely relies on morphology and emphasizes the lexical nature of the stress system. Prosodic specification in the morphology is favored for the following reasons: (i) It removes the need for features employed in traditional phonological analyses; (ii) Swedish has lexical specification of tone. Besides the prosodic specification in the morphology, there is a phonological stress rule that applies at the right edge of prosodic words. It should be noted, however, that phonological stress is assigned when there is no pre-assigned prosodic specification at that edge. One advantage of this combined approach is that there are no exceptions to the phonological algorithm. It should be emphasized that although a phonological stress rule exists, stress placement largely relies on morphologically specified prosodic information (Riad, 2014).

In this morphological approach, morphemes are categorized as (i) prosodically unspecified or (ii–iv) prosodically specified; prosodically specified morphemes are either (ii) lexically stressed, *tonic*, or (iii) occur in the positions before stressed syllables, *pretonic*, or (iv) after stressed syllables, *posttonic*. Roots are either (i) unspecified or (ii) tonic, whereas affixes may be any of the four categories. Some examples of the four categories are given in Table 1 (Riad, 2014).

**Table 1:** **Four prosodic types of morphemes: (i) unspecified, (ii) tonic (lexically stressed) (iii) posttonic (positioned after stressed syllables), and (iv) pretonic (positioned before stressed syllables)**.

**Prosodic specification**	**Example**
i. Unspecified	ka'lif
	ban-'al
ii. Tonic	'gris
	kub-'an
iii. Posttonic	'trev-lig
	'spel-are
iv. Pretonic	be-'löna
	för-'tala

Prosodically unspecified morphemes obtain stress through a phonological rule. The general phonological stress rule assigns stress to the rightmost available syllable, such as in *meka*ˈ*nik* ‘mechanics’ and *electrici*ˈ*tet* ‘electricity’. On the other hand, prosodically specified morphemes are argued to bear prosodic specifications, that is, being tonic, pretonic, and posttonic, as part of their lexical representations. These prosodic specifications directly determine stress placement^3^. It should be noted that phonological stress placement is sensitive to lexical prosodic information. For instance, if the rightmost syllable is a posttonic suffix, the last available syllable is the syllable preceding that suffix, as in *po*ˈ*lit-isk* ‘political’, where the root is unspecified and the suffix (–*isk*) is posttonic. In forms consisting of consecutive unspecified morphemes, stress is assigned to the rightmost morpheme, such as in *ba*ˈ*nal* ‘id’. and *banali*ˈ*tet* ‘banality’, where both the root and the suffix (–*itet*) are unspecified (Riad, 2012, 2014).

A way of diagnosing the prosodic specification of morphemes, suggested in Riad (2012, 2014), is to look at changes in vowel quality and vowel quantity between stressed and unstressed positions. In unspecified morphemes, if stress is moved away, the quality and quantity of vowels change radically, as in *politiker* [p℧ˈliːtıkεr] ‘politician’—*politisera* [p℧lıtıˈseːra] ‘to politicize’. On the other hand, in tonic morphemes, a trace of vowel length remains, and the vowel retains the same quality as seen in *slipa* [ˈsliːpa] ‘to grind’—*sliperi* [sliˑpeˈriː] ‘grindery’. Thus, unspecified morphemes retain no trace of stress if unstressed, whereas lexically stressed (tonic) morphemes retain traces of stress even after stress moves.

Similarly, it is suggested that lexical tone is part of the lexical specification of suffixes in Swedish; tones are typically associated with different suffixes rather than with the whole lexical item (Riad, 2009, 2012, 2014). Depending on the suffixes attached, the word stem receives either a low tone (Accent 1) or a high tone (Accent 2); for instance, a word like *lek*- ‘game’ is realized with an initial low tone if followed by a singular definite suffix –*en*, as in *lek-en* ‘the game’, whereas it receives a high tone if followed by a plural suffix –*ar*, as in *lek-ar* ‘games’ (Roll et al., 2013). It has been argued that while the association between high tones and suffixes is defined in the mental lexicon (marked), low tones are assigned postlexically (default) in Central Swedish (Riad, 2009, 2012, 2014, 2015)^4^. The electrophysiological effects of the lexical association of the tone with suffixes have been studied by investigating high and low stem tones with matching and mismatching suffixes (Roll et al., 2010, 2013). The aim of these studies was to examine if the stem tones function as cues for the associated suffixes and trigger an expectation for the following suffix information. As a result, they found a P600-like response for suffixes that typically correlate with high tone stems but were incorrectly preceded by a low stem tone, whereas no response was found for suffixes that typically correlate with low tone stems but were incorrectly preceded by a high stem tone. In summary, the authors interpreted these findings as evidence for the postlexical status of low tones and argued that there is a stronger association between high tone and its suffixes compared to low tones; high tone on a word stem is therefore claimed to cue the following suffix in online processing of Swedish.

Given that tone has been shown to be a part of the lexical specification of morphemes in Swedish with an electrophysiological approach and that lexical specification of prosodic features has been shown to be the case in various languages (Zora et al., 2015, 2016), the present study investigates whether stress is also a lexical property of morphemes in Swedish. The aim is to establish how lexically and phonologically stressed words are represented in the brain by using the mismatch negativity (MMN) component of event-related potentials (ERPs). MMN signals the brain's automatic reaction to any change in the auditory sensory input and is elicited irrespective of the subject's attention to the auditory stimulus (Näätänen et al., 1978, 2007; Näätänen and Winkler, 1999). In addition, MMN reflects the activation of long-term memory traces for lexical information (Dehaene-Lambertz, 1997; Pulvermüller et al., 2001; Shtyrov and Pulvermüller, 2002; Zora et al., 2015, 2016). Shtyrov and Pulvermüller (2002), for instance, investigated MMN responses elicited either by words presented among words or pseudowords or by pseudowords presented among words. Pseudowords differed from words only in the last phoneme (e.g., *bite* /bait/ and pseudoword /baip/). The findings indicated greater MMN response for words than for pseudowords, suggesting the existence of long-term memory traces for spoken words in the brain.

Memory traces for words are argued to be organized as strongly connected cell assemblies of cortical neurons, and these neural assemblies are fully activated when words are being processed (Pulvermüller, 1999; Pulvermüller et al., 2001). Therefore, in theory, presentation of a word that does not occur in the usual language output (either as a stem or a derivation) would not initiate the activation process. Memory traces for prosodic information and their contribution toward the lexical activation process have previously been investigated in English (Zora et al., 2015) and Turkish (Zora et al., 2016). The findings indicated that the memory traces for words are indeed activated on the sole basis of prosodic information, and the presence of prosodic representations in lexical representations has a differential effect on the processing of prosodic changes. Given these findings, the present study is the first to investigate whether changes in vowel quality (formant frequency) and in vowel quantity (duration) cue upcoming stress and activate potential lexical derivations.

If stress is a property of the stems defined in the mental lexicon, as argued in Riad (2012, 2014), changes in vowel quality and vowel quantity in lexically stressed words will in theory not activate memory traces associated with potential derivations because lexically stressed words will not undergo drastic changes in vowel quality and vowel quantity due to the stress shift. It was therefore predicted that changes in vowel quality and vowel quantity would cue upcoming stress and, hence, trigger memory traces for the potential derivations in phonologically stressed words, whereas they would only be considered as acoustic deviations in lexically stressed words. This hypothesis was investigated by inspecting MMN responses to changes in vowel quality and vowel quantity in phonologically and lexically stressed disyllabic words.

## Methods

### Participants

The participants were 15 native speakers of Swedish (9 females, 6 males; age range 22–57 years, *M* = 32.2, *SD* = 9.07). Handedness was assessed by the Edinburgh Handedness Inventory (Oldfield, 1971); all participants were right-handed. All were born and raised in Sweden, and reported normal development and hearing. Informed consent was signed prior to testing, and the study was approved by the Stockholm Regional Ethics Committee (2015/63-31).

### Stimuli

The material was a minimal pair, differing in the final segment: *banal* [baˈnɑːl] ‘id’. and *banan* [baˈnɑːn] ‘banana’. Both words are listed as basic vocabulary in the Frequency Dictionary of Present-Day Swedish (Allén, 1970). The words were pronounced in isolation by a female native speaker of Swedish (from Stockholm, 52 years old) in an anechoic chamber and were sampled at a rate of 44.1 kHz with 16 bits/per sample.

The choice of the stimuli was constrained by the prosodic specification of words. Despite extensive searching in dictionaries and through a lexical database for Swedish developed by Nordisk Språkteknologi (NST)^5^, the *banal*—*banan* pair was, to our knowledge, the only minimal pair that enabled the diagnostic of prosodic specification^6^. In order to confirm the prosodic specification of these words, the unspecified morphemes –*itet* and –*eri* were added to the stems, as in *banalitet* [banalıˈteːt] ‘banality’ and *bananeri* [banɑˑnεˈriː], and vowel quality and vowel quantity changes were scrutinized. Although it is not a lexical entry in Swedish dictionaries, *bananeri* is not considered an absolute nonsense word due to the productivity of the suffix –*eri*, which can be combined with any root, typically to denote an ongoing activity or a place where an activity takes place (Riad, 2012). Swedish speakers apparently relate *bananeri* to a representation similar to that of *orangeri* ‘orangery’, a protected place, especially a greenhouse, for growing oranges in cool climates.

Table 2 illustrates the acoustic effects in the vowel of the second syllable as stress shifted to the last syllable after derivational morphemes were attached. Vowel quality and vowel quantity measurements were performed in Praat (Boersma and Weenink, 2014). The vowel onset and offset were determined based on the information from waveforms and spectrograms. Table 2 presents the duration (in ms), overall intensity (dB) and frequency measures for F0, F1, and F2 (in Hz) over the vowel of the second syllable in *banan* and *banal*, and in their derivations *bananeri* and *banalitet*, as well as in the pseudoword *sananitet*.

**Table 2:** **Vowel durations (in ms), overall intensity (in dB), and frequency measures for F0, F1, and F2 (in Hz) over the vowel of the second syllable in the phonologically stressed word *banal* and the lexically stressed word *banan* and in their potential derivations *banalitet* and *bananeri*, as well as in the pseudoword *sananitet* (vowel marked in bold)**.

	baˈnɑːl	ban**a**l**I**ˈteːt	baˈnɑːn	banɑˑnεˈriː	san**a**n**I**ˈteːt^*^
/a/ - σ2 Duration in ms	350	119	337	158	112
/a/ - σ2 Intensity in dB	72	74	73	73	74
/a/ - σ2 F0 in Hz	161	154	160	158	151
/a/ - σ2 F1 in Hz	675	611	601	631	654
/a/ - σ2 F2 in Hz	1086	1479	1089	1194	1396

* Pseudoword.

As seen in Table 2, while varying quite distinctly in *banalitet*, the vowel retained nearly the same quality in *bananeri* even after the stress moved. Moreover, in contrast to *banalitet*, some traces of vowel length remained in *bananeri*. In short, the second vowel in *banalitet* got shorter and relatively more close and fronted compared to the second vowel in *banal*. It should be noted that the most drastic change was F2-related. These acoustic effects indicated *banal* to be a phonologically stressed word and *banan* as a lexically stressed word. Table 2 provides further information about the phonetics of Swedish lexical stress: F0 and duration were employed in marking the stressed syllable, whereas overall intensity did not lead to any straightforward interpretation. This is in line with previous findings that identified duration and F0 as correlates, with duration being the primary correlate, and which argued that intensity is not a consistent correlate of stress in Swedish (Fant and Kruckenberg, 1994; Eriksson et al., 2013).

The experiment consisted of two blocks: A phonologically stressed word block (PSW) and a lexically stressed word block (LSW). In a passive oddball paradigm, the frequently presented standards [banɑːl] and [banɑːn], were occasionally replaced by deviants [banal] and [banan], respectively, in the two blocks (Audio Files 1–4 are provided as Supplementary Material to this article). The deviants differed from the standards in vowel quality and vowel quantity on the second syllable. It should be noted that the deviants do not occur in actual spoken Swedish.

**Figure 1:**
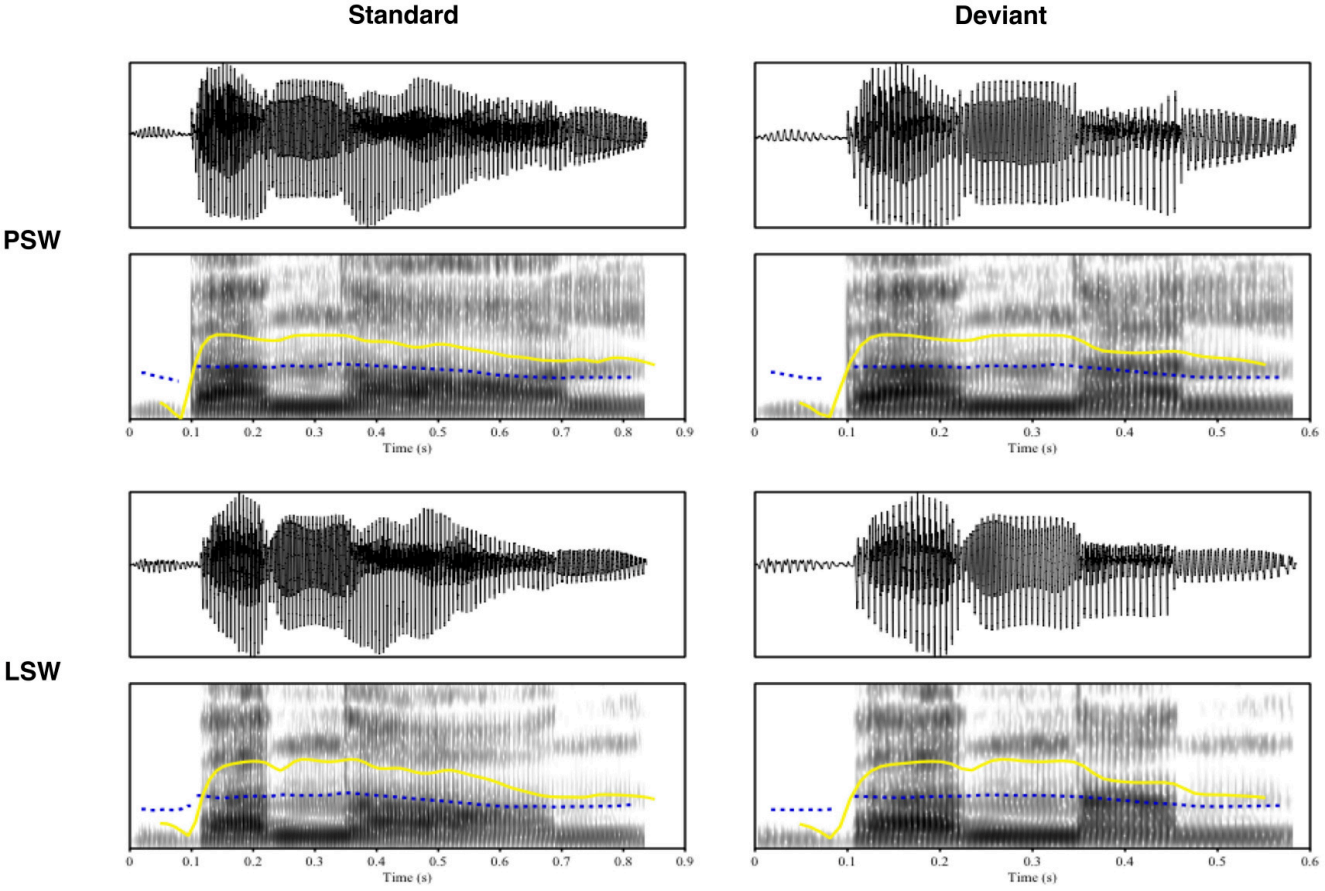
**Waveforms and spectrograms of the standard and deviant stimuli in each block**. Blue dotted line, Pitch; Yellow solid line, Intensity; PSW, Phonologically stressed word; LSW, Lexically stressed word.

In order to keep the difference minimal across the stimuli, the deviants were created out of the standards through a cross-splicing technique. The critical vowels (i.e., second vowels) in [banɑːl] and [banɑːn] were extracted and replaced with the critical vowels in [banalıˈteːt] and [sananıˈteːt]^*^ to produce [banal] and [banan]. An experienced phonetician was asked to pronounce the pseudoword [sananıˈteːt]^7^ in order to get an [a] to be spliced in [banan] (Table 2). In this way, it was ensured that the replacement [a] was extracted from the same surrounding segmental context as the standards. All stimuli were matched for total length: The standard stimuli were ~830 ms in duration, and the deviant stimuli ~580 ms. The onset of the second vowel was at ~350 ms. The intensity was normalized and the F0 contour was flattened and approximated across the standards and deviants to increase listeners' reliance on duration and first/second formant frequencies. Table 3 presents the duration (in ms), overall intensity (in dB) and frequency measures for F0, F1, and F2 (in Hz) over the vowel of the second syllable in the standard and deviant stimuli in each block. Figure 1 presents the waveforms and spectrograms of the standard and deviant stimuli in each block.

**Table 3:** **Vowel durations (in ms), overall intensity (in dB), and frequency measures for F0, F1, and F2 (in Hz) over the vowel of the second syllable of standards and deviants in the phonologically stressed word block (PSW) and the lexically stressed word block (LSW) (vowel marked in bold)**.

	**PSW**		**LSW**	
	**Standard**	**Deviant**	**Standard**	**Deviant**
	baˈnɑːl	ban**a**l	baˈnɑːn	ban**a**n
/a/ - σ2 Duration in ms	350	114	337	103
/a/ - σ2 Intensity in dB	71	71	71	71
/a/ - σ2 F0 in Hz	145	145	145	145
/a/ - σ2 F1 in Hz	675	621	601	671
/a/ - σ2 F2 in Hz	1086	1495	1089	1425

As mentioned above, when stress moves, vowel quality and vowel quantity varies distinctly in phonologically stressed words but not in lexically stressed words. Considering potential lexical derivations, e.g., *banalitet* [banalıˈteːt] and *bananeri* [banɑˑnεˈriː], the deviant [banal] is acceptable for phonologically stressed words, but [banan] is unacceptable for lexically stressed words. A greater MMN response is therefore predicted for deviants in PSW than in LSW due to strongly interconnected neuronal networks. Since vowel quality and quantity changes are associated with morphological derivations in PSW, neural networks for these derivations would be activated only in PSW. Vowel quality and quantity changes would therefore elicit greater negativity in PSW than LSW, which lacks such an association between vowel changes and derivations.

### Procedure

The experiment was run using E-Prime (version 2.0). The stimuli were delivered via loudspeakers at a comfortable listening level of 60–65 dB at source. The stimuli were presented in an auditory oddball paradigm: Standard stimulus (*p* = 8/10) was randomly replaced by deviant stimulus (*p* = 2/10). The stimulus onset asynchrony (SOA) was set at 1200 ms. The experiment had two blocks, PSW and LSW, each block consisting of 640 standards and 160 deviants. Each block took 16 min, and the participants were given a chance to take a break between the blocks. The order of the blocks was counterbalanced across the participants. A silent documentary was used to take participants' attention off the auditory stimuli.

### Electroencephalography recordings and data analysis

The electroencephalography (EEG) data were collected at a sampling rate of 2048 Hz using the BioSemi ActiveTwo system and ActiView acquisition software (BioSemi, Netherlands) from 16 electrodes. In all, 7 external electrodes were used: 4 for monitoring horizontal and vertical eye movement, 2 for mastoid recordings and 1 for nose recording. Offline data analysis was carried out in MATLAB (The Math Works Inc., Natick, Massachusetts, USA) using the EEGLAB toolbox (Delorme and Makeig, 2004). The continuous EEG data were first resampled to 256 Hz and band-pass filtered at 0.5–30 Hz. The BioSemi enables referencing the data for online EEG display without actually referencing the data. Referencing is accomplished offline; in this study, the signals were referenced offline to the nose channel. To identify and remove eye artifacts, independent component analysis (ICA; Jung et al., 2000) was carried out. The EEG data were then segmented into epochs from −100 to 900 ms with a time window of 100 ms for the baseline correction. ERPs were time-locked to the word onset. Artifact rejection was set to remove activation exceeding ±100 μV at any channel. The grand average was computed per stimulus type for all participants, and deviant-minus-standard subtractions were calculated for the deviants.

### Statistical analysis

Statistical analysis was carried out using SPSS (International Business Machines Corp., Armonk, New York, USA). The electrodes were grouped together in three regions of interest (ROI), each having three electrodes: frontal (F3, FZ, F4), central (C3, CZ, C4), and parietal (P3, PZ, P4). The measurement window was determined by visual inspection of grand average ERP waveforms. Amplitudes were computed as a mean voltage within a 60-ms-window centered around the peak.

Four-way repeated-measures ANOVA with factors of Time (two levels: first and second time window), ROI (three levels: frontal, central and parietal), Block (two levels: PSW and LSW) and Stimuli (two levels: standard and deviant) were performed. If significant interactions occurred, follow-up ANOVAs were performed, and the levels were then compared in pairwise comparisons. Additional two-tailed *t*-tests were carried out to compare the amplitudes obtained by deviant-minus-standard subtractions in PSW against those in LSW in each ROI and in each time window. Mean values were reported with standard deviations. *P*-values were given with Greenhouse-Geisser correction in case of sphericity violations. Effect sizes were reported with η^2^ (partial η^2^).

## Results

**Figure 2:**
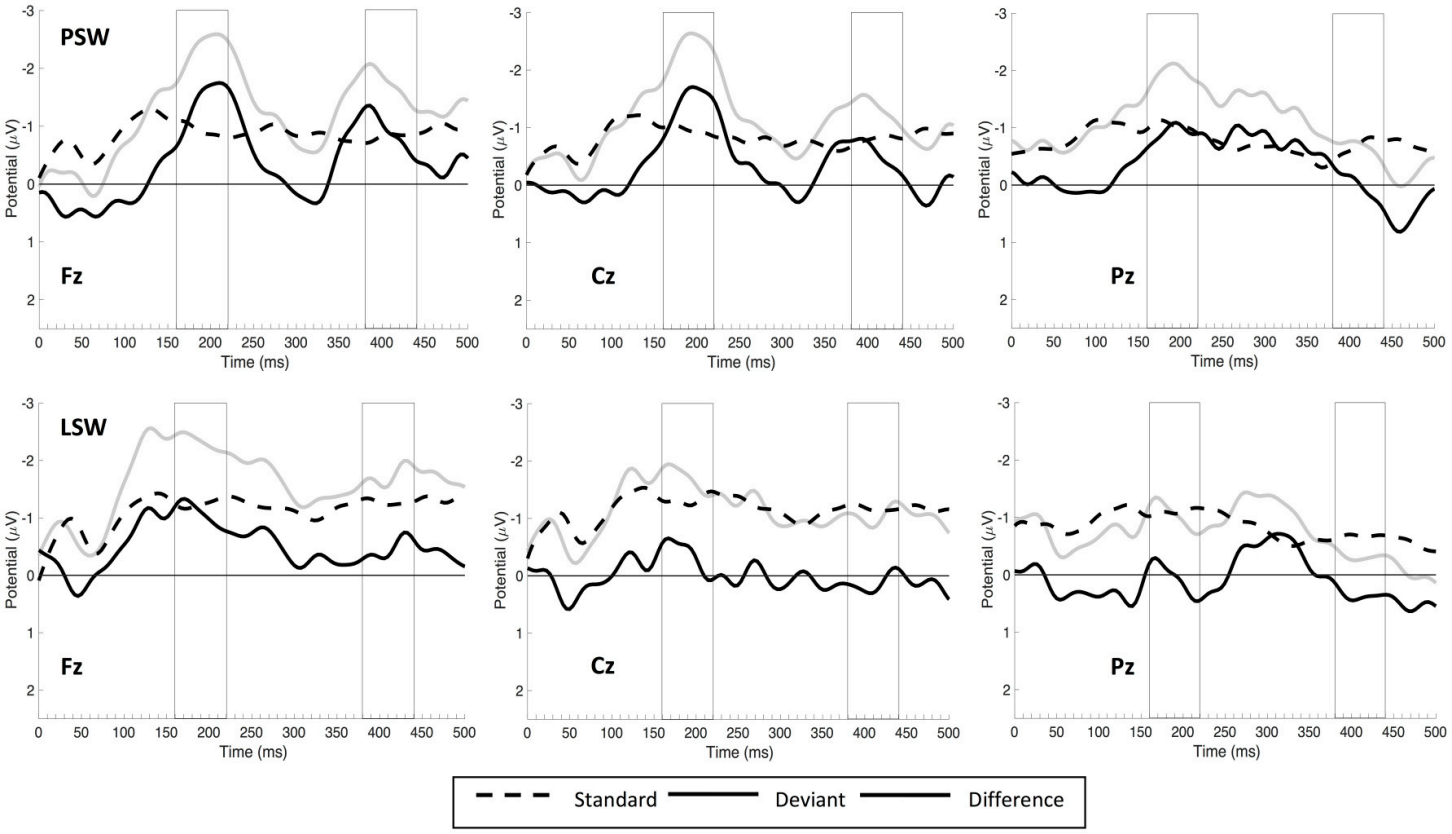
**The grand average of standard, deviant and difference (deviant-minus-standard) waveforms recorded from Fz, Cz, and Pz in PSW and LSW blocks**. Negativity is plotted upward. PSW, Phonologically stressed word; LSW, Lexically stressed word.

**Figure 3:**
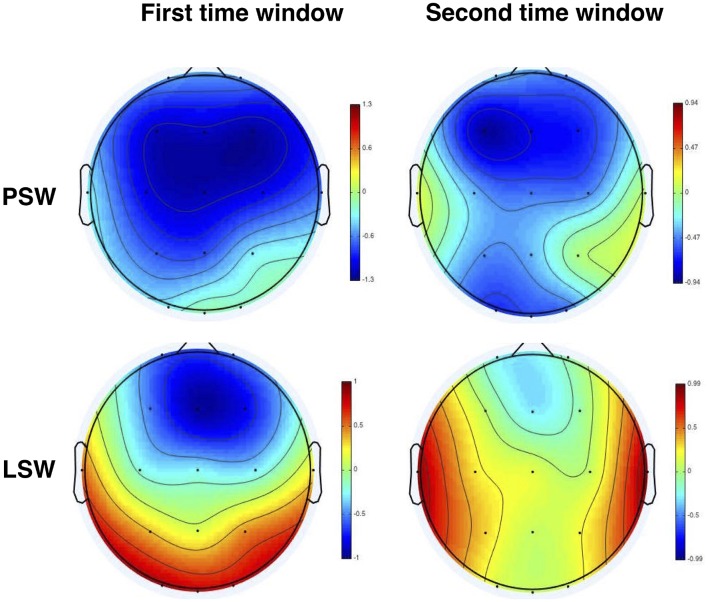
**Topographic difference maps at the first time window (150–250 ms) and at the second time window (350–450 ms) in PSW and LSW blocks**. PSW, Phonologically stressed word; LSW, Lexically stressed word.

The grand average of standard, deviant and difference waveforms recorded from Fz, Cz and Pz in PSW and LSW blocks are shown in Figure 2,^8^. The deviants seem to elicit two MMN responses: one with a time-course of 150–250 ms, and another with a time-course of 350–450 ms. While there are two clear MMN peaks in PSW, only the first peak appears to be pronounced in LSW. Figure 3 shows topographic difference maps at the first and second time window for PSW and LSW blocks.

**Figure 4:**
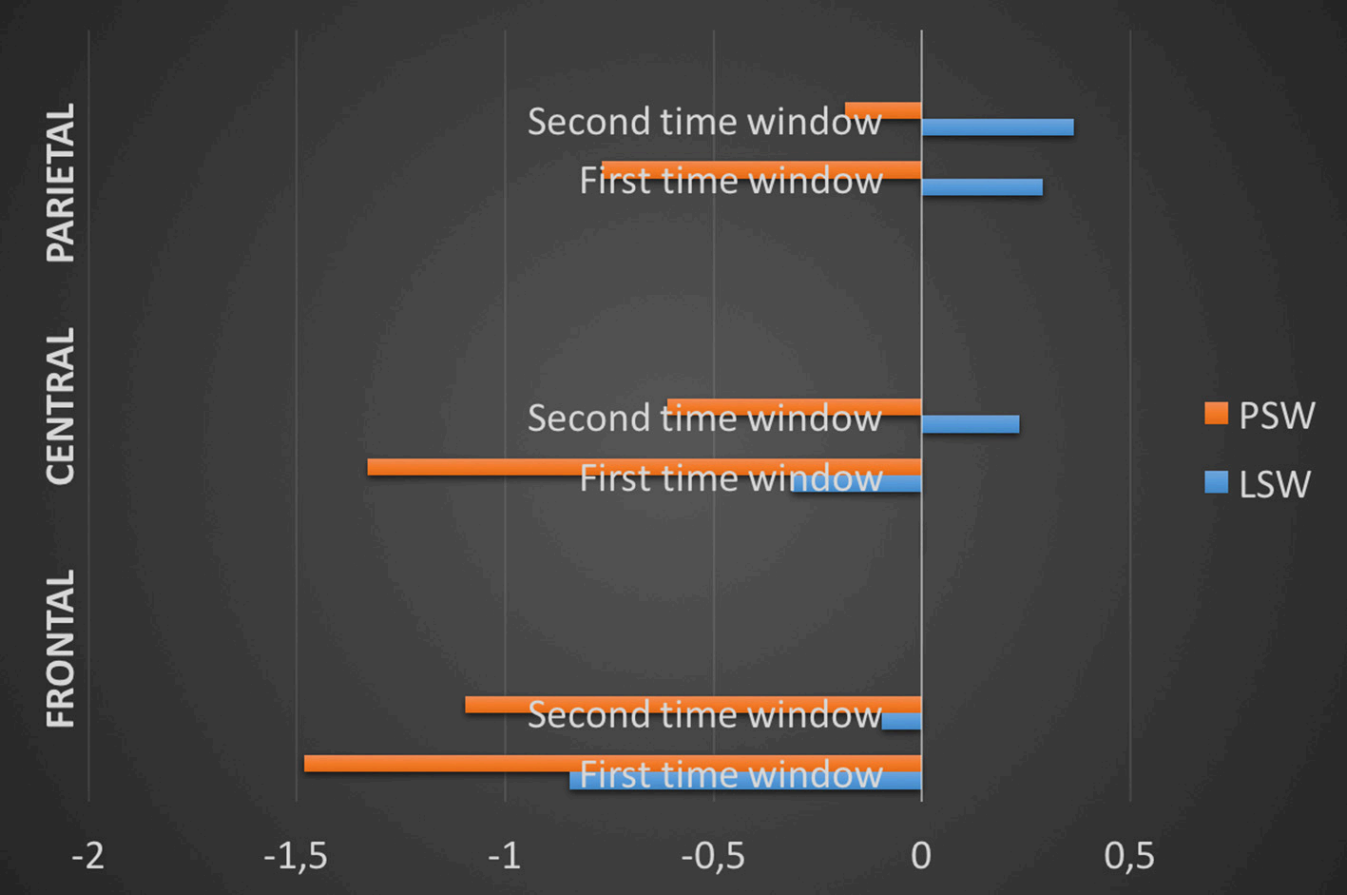
**Mean deviant-minus-standard subtractions in each time window and in each ROI per block**. ROI, Region of interest; PSW, Phonologically stressed word; LSW, Lexically stressed word.

Data entered for statistical analysis was computed from time windows 160–220 ms and 380–440 ms. The minimum number of accepted standard and deviant items per participant was 541 and 135 in LSW and 523 and 137 in PSW, respectively. Figure 4 indicates mean deviant-minus-standard subtractions in each time window and in each ROI per block.

The four-way repeated measures ANOVA indicated a significant main effect of Time [*F*_(1, 14)_ = 18.419, *p* = 0.001, η^2^ = 0.568]; a significant main effect of ROI [*F*_(2, 28)_ = 5.045, *p* = 0.032, η^2^ = 0.265]; a significant two-way interaction of ROI with Stimuli [*F*_(2, 28)_ = 8.485, *p* = 0.001, η^2^ = 0.377]; a significant two-way interaction of Time with Stimuli [*F*_(1, 14)_ = 7.947, *p* = 0.014, η^2^ = 0.362]; a significant two-way interaction of Block with Stimuli [*F*_(1, 14)_ = 11.987, *p* = 0.004, η^2^ = 0.461]; a significant three-way interaction of Time with ROI and Block [*F*_(2, 28)_ = 8.621, *p* = 0.001, η^2^ = 0.381]; and a four-way interaction of Time with ROI, Block and Stimuli [*F*_(2, 28)_ = 7.183, *p* = 0.011, η^2^ = 0.339; Table 4].

**Table 4:** **Results for four-way repeated measures ANOVA with factors of Time window (Time), Regions of interest (ROI), Block and Stimuli (Stim)**.

**Factor**	***F***	***p***	**η^2^**
**FOUR-WAY REPEATED MEASURES ANOVA**			
Time	*F*_(1, 14)_ = 18.419	0.001^*^	0.568
ROI	*F*_(2, 28)_ = 5.045	0.032^*^	0.265
Block	*F*_(1, 14)_ = 0.004	0.951	0.000
Stim	*F*_(1, 14)_ = 2.508	0.136	0.152
Time × ROI	*F*_(2, 28)_ = 1.806	0.196	0.114
Time × Block	*F*_(1, 14)_ = 0.357	0.559	0.025
ROI × Block	*F*_(2, 28)_ = 2.412	0.134	0.147
ROI × Stim	*F*_(2, 28)_ = 8.485	0.001^*^	0.377
Time × Stim	*F*_(1, 14)_ = 7.947	0.014^*^	0.362
Block × Stim	*F*_(1, 14)_ = 11.987	0.004^*^	0.461
Time × ROI × Block	*F*_(2, 28)_ = 8.621	0.001^*^	0.381
Time × ROI × Stim	*F*_(2, 28)_ = 3.719	0.062	0.210
Time × Block × Stim	*F*_(1, 14)_ = 0.232	0.638	0.016
ROI × Block × Stim	*F*_(2, 28)_ = 0.586	0.563	0.040
Time × ROI × Block × Stim	*F*_(2, 28)_ = 7.183	0.011^*^	0.339

* p < 0.05.

Follow-up three-way repeated measures ANOVAs were carried out to investigate the interaction of Time with ROI and Stimuli in each block (Follow-up ANOVA 1, Table 5). In LSW, there was a significant main effect of Time [*F*_(1, 14)_ = 11.376, *p* = 0.005, η^2^ = 0.448]; a significant main effect of ROI [*F*_(2, 28)_ = 5.915, *p* = 0.023, η^2^ = 0.297]; a significant two-way interaction of ROI with Stimuli [*F*_(2, 28)_ = 7.053, *p* = 0.003, η^2^ = 0.335]; and a significant three-way interaction of Time with ROI and Stimuli [*F*_(2, 28)_ = 9.977, *p* = 0.004, η^2^ = 0.416]. In PSW, there was a significant main effect of Time [*F*_(1, 14)_ = 12.823, *p* = 0.003, η^2^ = 0.478]; a significant main effect of Stimuli [*F*_(1, 14)_ = 5.258, *p* = 0.038, η^2^ = 0.273]; a significant two-way interaction of Time with ROI [*F*_(2, 28)_ = 4.935, *p* = 0.015, η^2^ = 0.261]; and a significant two-way interaction of ROI with Stimuli [*F*_(2, 28)_ = 4.499, *p* = 0.020, η^2^ = 0.243]. This follow-up analysis indicated that the interaction of Time with ROI and Stimuli was significant only in LSW.

**Table 5:** **Results for follow-up three-way repeated measure ANOVAs**.

**Block**	**Factor**	***F***	***p***	**η^2^**
**FOLLOW-UP ANOVA 1**				
LSW	Time	*F*_(1, 14)_ = 11.376	0.005^*^	0.448
	ROI	*F*_(2, 28)_ = 5.915	0.023^*^	0.297
	Stim	*F*_(1, 14)_ = 0.214	0.650	0.015
	Time × ROI	*F*_(2, 28)_ = 0.062	0.868	0.004
	Time × Stim	*F*_(1, 14)_ = 4.428	0.054	0.240
	ROI × Stim	*F*_(2, 28)_ = 7.053	0.003^*^	0.335
	Time × ROI × Stim	*F*_(2, 28)_ = 9.977	0.004^*^	0.416
PSW	Time	*F*_(1, 14)_ = 12.823	0.003^*^	0.478
	ROI	*F*_(2, 28)_ = 2.870	0.099	0.170
	Stim	*F*_(1, 14)_ = 5.258	0.038^*^	0.273
	Time × ROI	*F*_(2, 28)_ = 4.935	0.015^*^	0.261
	Time × Stim	*F*_(1, 14)_ = 3.294	0.091	0.190
	ROI × Stim	*F*_(2, 28)_ = 4.499	0.020^*^	0.243
	Time × ROI × Stim	*F*_(2, 28)_ = 0.377	0.266	0.090

Interaction of Time with Regions of interest (ROI) and Stimuli (Stim) in the phonologically stressed word block (PSW) and the lexically stressed word block (LSW).

* p < 0.05.

Follow-up two-way repeated measures ANOVAs were carried out for the three-way interaction in LSW; the interaction of Time and Stimuli was scrutinized in each ROI (Follow-up ANOVA 2, Table 6). In frontal sites, there was a significant main effect of Time [*F*_(1, 14)_ = 9.050, *p* = 0.009, η^2^ = 0.393] and a significant two-way interaction of Time with Stimuli [*F*_(1, 14)_ = 10.722, *p* = 0.006, η^2^ = 0.434]. In central sites, there was a significant main effect of Time [*F*_(1, 14)_ = 9.827, *p* = 0.007, η^2^ = 0.412] and a significant two-way interaction of Time with Stimuli [*F*_(1, 14)_ = 6.966, *p* = 0.019, η^2^ = 0.332]. In parietal sites, there was a significant main effect of Time [*F*_(1, 14)_ = 8.456, *p* = 0.011, η^2^ = 0.377]. Follow-up analyses in frontal and central sites indicated that the difference between standard (*M* = −1.192 μV, *SD* = 0.284) and deviant (*M* = −2.109 μV, *SD* = 0.545) was significant only in frontal sites (*p* = 0.019) and only in the first time window. These analyses indicated that the MMN response was restricted to the first time window and to the frontal sites in LSW.

Table 6**Results for follow-up two-way repeated measures ANOVAs**.

**Table:** 

**ROI**	**Factor**	***F***	***p***	**η^2^**
**FOLLOW-UP ANOVA 2**				
Frontal	Time	*F*_(1, 14)_ = 9.050	0.009^*^	0.393
	Stim	*F*_(1, 14)_ = 2.753	0.119	0.164
	Time × Stim	*F*_(1, 14)_ = 10.722	0.006^*^	0.434
Central	Time	*F*_(1, 14)_ = 9.827	0.007^*^	0.412
	Stim	*F*_(1, 14)_ = 0.066	0.800	0.005
	Time × Stim	*F*_(1, 14)_ = 6.966	0.019^*^	0.332
Parietal	Time	*F*_(1, 14)_ = 8.456	0.011^*^	0.377
	Stim	*F*_(1, 14)_ = 0.906	0.357	0.061
	Time × Stim	*F*_(1, 14)_ = 0.001	0.972	0.000

**Table:** 

**ROI**	**Time window**	**Factor**	***F***	***p***	**η^2^**	**Stim level**	***M***	***SD***
Frontal	1st	Stim	*F*_(1, 14)_ = 6.964	0.019^*^	0.332	Standard	−1.192	0.284
						Deviant	−2.109	0.545
	2nd	Stim	*F*_(1, 14)_ = 0.051	0.825	0.004	Standard	−1.175	0.286
						Deviant	−1.242	0.501
Central	1st	Stim	*F*_(1, 14)_ = 1.531	0.236	0.099	Standard	−1.270	0.252
						Deviant	−1.655	0.481
	2nd	Stim	*F*_(1, 14)_ = 0.445	0.515	0.031	Standard	−1.156	0.228
						Deviant	−0.929	0.464

Interaction of Time with Stimuli (Stim) in each of the Regions of interest (ROI) in the lexically stressed word block (LSW).

* p < 0.05.

In order to assess the MMN responses in PSW block, a follow-up analysis to two-way interaction of ROI with Stimuli was carried out (Table 7). The difference between standard (*M* = −0.741 μV, *SD* = 0.230) and deviant (*M* = −1.781 μV, *SD* = 0.494) was significant in frontal sites (*p* = 0.009); the difference between standard (*M* = −0.808 μV, *SD* = 0.194) and deviant (*M* = −1.639 μV, *SD* = 0.455) was also significant in central sites (*p* = 0.027). The analysis indicated that regardless of time window, there were significant MMN responses in both frontal and central sites in PSW.

**Table 7:** **Follow-up analysis to two-way interaction of Regions of interest (ROI) with Stimuli (Stim) in the phonologically stressed word block (PSW)**.

**ROI**	**Factor**	***F***	***p***	**η^2^**	**Stim level**	***M***	***SD***
Frontal	Stim	*F*_(1, 14)_ = 9.157	0.009^*^	0.395	Standard	−0.741	0.230
					Deviant	−1.781	0.494
Central	Stim	*F*_(1, 14)_ = 6.127	0.027^*^	0.304	Standard	−0.808	0.194
					Deviant	−1.639	0.455
Parietal	Stim	*F*_(1, 14)_ = 1.665	0.218	0.106	Standard	−0.582	0.154
					Deviant	−1.101	0.495

* p < 0.05.

Additional two-tailed *t*-tests were carried out to compare the amplitudes obtained by deviant-minus-standard subtractions in PSW against those in LSW in each ROI and in each time window (Table 8). In the first time window, the difference between PSW (*M* = −1.136 μV, *SD* = 1.586) and LSW (*M* = −0.916 μV, *SD* = 1.345) did not reach significance at frontal sites (*p* = 0.430); at central sites, PSW (*M* = −1.113 μV, *SD* = 1.622) elicited greater negativity than LSW (*M* = −0.384 μV, *SD* = 1.204, *p* = 0.022); at parietal sites, PSW (*M* = −0.656 μV, *SD* = 1.644) elicited negativity, whereas LSW elicited positivity (*M* = 0.210 μV, *SD* = 0.915, *p* = 0.015). In the second time window, at frontal sites, PSW (*M* = −0.943 μV, *SD* = 1.218) elicited greater negativity than LSW (*M* = −0.067 μV, *SD* = 1.153, *p* = 0.006); at central sites, PSW (*M* = −0.548 μV, *SD* = 1.118) elicited negativity, whereas LSW elicited positivity (*M* = 0.227 μV, *SD* = 1.320, *p* = 0.009); the difference between PSW (*M* = −0.382 μV, *SD* = 1.553) and LSW (*M* = 0.220 μV, *SD* = 1.136) did not reach significance at parietal sites (*p* = 0.073).

**Table 8:** **Two-tailed *t*-tests with amplitudes obtained from deviant-minus-standard subtractions in the phonologically stressed word block (PSW) and the lexically stressed word block (LSW) in each Time window and in each of the Regions of interest (ROI)**.

**Time window**	**ROI**	***p***
1st time window	Frontal	0.430
	Central	0.022^*^
	Parietal	0.015^*^
2nd time window	Frontal	0.006^*^
	Central	0.009^*^
	Parietal	0.073

* p < 0.05.

Taken together, these results indicate that vowel quality and quantity changes elicit MMN responses at around 160 ms after change onset, confirming automatic response of the brain to a change in auditory sensory input, which typically peaks at 150–250 ms from change onset (Näätänen et al., 2007). The findings further indicate that the MMN responses are maximal over frontal and central scalp locations, in line with previous findings (Näätänen and Winkler, 1999; Näätänen et al., 2007). The processing differences between PSW and LSW are reflected in the amplitude and topography of MMN responses. In LSW, the MMN response is restricted to the first time window and to the frontal sites; in the second time window, the neural response to LSW develops into a positive response. On the other hand, in PSW, the MMN response is present in both time windows, and in central sites as well as in frontal sites. In short, the amplitude of MMN is greater for PSW than it is for LSW, and the topographic distribution of MMN response is more widespread for PSW than for LSW.

## Discussion

By recording neural responses to changes in vowel quality and vowel quantity, the present study has investigated the lexical specification of stress information in Swedish. The neural responses to formant frequency and duration changes in both phonologically and lexically stressed words were recorded. Given that only phonologically assigned stress varies with the phonological shape, it was predicted that formant frequency and duration changes would activate assembly-internal connections and cue upcoming stress and, hence, lexical derivation only in phonologically stressed words. If morphologically determined stress is defined in the mental lexicon and traces of it are stable even after stress moves, as argued in Riad (2012, 2014), formant frequency and duration changes will in theory not activate memory traces associated with potential derivations in lexically stressed words.

The choice of the stimuli was constrained by the prosodic specification of words. The *banal*—*banan* pair is, to our knowledge, the only minimal pair that can be used to make a distinction between phonological and lexical specification. Prosodic classification was confirmed by examining changes in vowel quality and vowel quantity after adding unspecified morphemes to the stems, as in *banalitet*—*bananeri*. The drawback of this pair is that *bananeri*, the potential derivation of *banan*, is not a real word; that is, it does not have a lexical entry in Swedish dictionaries. However, Swedish speakers do not consider *bananeri* to be an absolute nonsense word. It should also be emphasized that the lexical status of *bananeri* is not central to the present study; *bananeri* is employed to confirm the prosodic classification of *banan*. Vowel quality and quantity changes are associated with morphological derivations only in the phonologically stressed word *banal*. Vowel quality and quantity changes are not expected in *banan* whatever the potential derivation is (either *bananeri* or another word). That is, *banan* lacks an association between vowel changes and potential derivations, and therefore, there cannot be any memory traces associated with vowel changes in *banan*.

One might question the appropriateness of choosing the only existing minimal pair attesting to this morpho-phonological phenomenon in Swedish. However, the validity of this phenomenon does not depend on the existence/plurality of minimal pairs (for a list of specified and unspecified morphemes and examples, see Riad, 2014). The motivation for having a minimal pair was due to the experimental paradigm employed in the present study; since the MMN paradigm is sensitive to physical changes, it was crucial to control the physical characteristics of the stimuli and, thus, have a minimal pair. It should be possible to investigate this phenomenon with another experimental paradigm that is not limited to individual language stimuli. However, it should be noted that it is important to reduce stimulus variance in order to study the early effects of word processing, and it is, therefore, more appropriate to use single words rather than a large group of stimuli (Pulvermüller and Shtyrov, 2006).

The findings of the present study demonstrate that the brain does not only detect vowel quality and vowel quantity changes, but also uses them to activate assembly-internal connections; there is a difference in how the brain treats vowel quality and vowel quantity changes depending on the prosodic specification of words. First of all, the topographic distribution of MMN responses is more widespread for phonologically stressed words than for lexically stressed words, which demonstrates that a larger area is recruited for the processing of vowel changes in phonologically stressed words than in lexically stressed words. This finding is not surprising given that vowel changes are associated with potential derivations only in phonologically stressed words, whereas lexically stressed words lack such an association. Strongly connected cell assemblies and the activation of these assemblies for vowel changes associated with potential derivations explain the difference in this topographic distribution. Secondly, the MMNs to vowel quality and vowel quantity changes are obtained in two time windows for phonologically stressed words, while restricted to one time window for lexically stressed words. In the first time window, both phonologically stressed and lexically stressed words elicit MMN responses; the amplitude is, however, smaller in lexically stressed words than in phonologically stressed words. In the second time window, the negative response is stable in phonologically stressed words, while developing into a positive response in lexically stressed words, which might indicate attention orientation to acoustic change. Alternatively, one could argue the second negativity in phonologically stressed words to be a Late Discriminative Negativity (LDN). Similar to MMN, LDN (also called late MMN) is elicited by deviant stimuli in a passive oddball paradigm and shows a fronto-central distribution. Although its functional significance remains unclear, LDN has often been associated with higher cognitive processes such as long-term memory, similar to MMN. However, LDN typically occurs in a later time window (400–700 ms after change onset) and is found in infants and children (Cheour et al., 2001; Mueller et al., 2008). Given that the functional significance of LDN remains unclear and it is typically present in infants, the MMN interpretation has been adopted for the second negativity in the present study. It should, however, be noted that whatever interpretation is taken, either MMN or LDN, this negative response is apparently related to long-term memory traces for vowel changes associated with potential derivations.

Given that memory traces for words are organized as strongly connected cell assemblies and these neural assemblies are fully activated when words are being processed (Pulvermüller, 1999; Pulvermüller et al., 2001), salient changes in vowel quality and vowel quantity are expected to trigger potential lexical derivations; that is, changes in vowel quality and vowel quantity would signal upcoming stress and, hence, addition of a morpheme at the end of the form. Memory traces associated with vowel quality and vowel quantity changes are therefore activated in phonologically stressed words, since phonologically assigned stress varies with the phonological shape. However, presentation of a word that does not occur in the usual language output (either as a stem or a derivation) would not initiate the activation process. Changes in vowel quality and vowel quantity do not therefore activate memory traces in lexically stressed words since they do not undergo drastic vowel quality and vowel quantity changes even after stress moves.

The present findings make a significant contribution to the literature by demonstrating the involvement of morphology in the Swedish stress system. As mentioned in the introduction, like those of many other Germanic languages, the stress system of Swedish has mostly been treated phonologically; recently, however, researchers have begun to recognize the central role of morphology in these systems. The advantage of giving morphological information a central role is that it removes the need for features employed in traditional phonological analyses. Furthermore, there are some circumstances that favor morphological stress generalizations over automatic phonological generalizations in Swedish. First of all, there is a weaker sense of automaticity to the stress system in Swedish in contrast to the more prototypically phonological stress systems such as French or Finnish^9^. For instance, the stress rule in French and Finnish strongly influences speakers' pronunciation of foreign languages. Swedish speakers, on the other hand, do not encounter difficulties pronouncing words with different stress patterns; however, they cannot easily abandon postlexical generalizations of Swedish phonology. This leads to the conclusion that, in comparison with Finnish and French, the phonological part of the Swedish stress system is very restricted, and stress placement is mostly determined by morphological marking. Secondly, words that are loaned to Swedish tend to retain the stress of the source language. If there were a strong, automatic phonological generalization for stress placement, it would have influenced at least some of these loanwords. However, this is not the case in Swedish, which again favors an analysis based on morphological stress generalizations (Riad, 2014).

In sum, the present study is the first EEG study to indicate the direct involvement of morphology in the Swedish stress system. The findings indicate that the presence of lexical stress is inferred from vowel quality and vowel quantity; changes in phonological shape due to stress shift cue the upcoming stress and the potential derivations. However, these findings need to be supported by further research. As a follow up, a group of listeners who know no Swedish will be tested to see if they differ from native speakers in their processing of these changes of vowel quality and quantity in phonologically and lexically stressed words. This would enable the comparison of native and nonnative processing, and strengthen the interpretation of the present study regarding the activation of neural assemblies associated with vowel changes and potential derivations. Moreover, investigating the derived versions, for instance, *bananeri* and *banalitet*, will provide greater detail on the lexical specification of vowel quality and quantity information. The mismatch of vowel quality and vowel quantity in phonologically and lexically stressed words, as in [banalıˈteːt] vs. [banɑˑlıˈteːt]^*^ and [banɑˑnεˈriː] vs. [bananeˈriː]^*^, might result in different neural responses. Given that the present study has indicated the involvement of morphology, future research should shed more light on lexical specification of prosodic information in Swedish.

## Author contributions

HZ, TR, IS, MH: The conception and design of the work; drafting the work; final approval of the version to be published.

### Conflict of interest statement

The authors declare that the research was conducted in the absence of any commercial or financial relationships that could be construed as a potential conflict of interest.

## Footnotes

^1^By *tonal accent system*, it is meant that there is a phonological distinction between two tonal configurations, and a lexical distinction is attributed to the prominence function of these tonal configurations. There are two accent patterns in Swedish: *Accent 1* and *Accent 2* (Heldner, 2001; Riad, 2014).

^2^Ignoring a particular segment for stress placement.

^3^It is crucial to note that no stress is assigned by affixes; that is, affixes are not pre- or post-stressing but are rather pre- or post-tonic (Riad, 2012).

^4^There are opposing views regarding the markedness of tonal accents in Scandinavian tonal dialects. In contrast to the above-mentioned view, other researchers argue that Accent 1 is marked, while Accent 2 is the default (for Swedish, see Lahiri et al., 2005, 2006; Felder et al., 2009, and for Norwegian, see Wetterlin, 2010).

^5^http://www.nb.no/sprakbanken.

^6^Minimal pairs are hard to find since tonic morphemes are usually stressed on the root-initial syllable (mainly etymologically Germanic), whereas phonologically stressed words tend to have unstressed syllables preceding the stressed syllable (mainly etymologically Romance or Greek) (Riad, 2012, 2014).

^7^Splicing [a] from [banaltet] to [banan] would be problematic due to the segmental differences surrounding the vowel.

^8^The onset of the second vowel was used as the zero point in figures given that the standards and deviants were identical up to the second vowel and information about any difference between the standards and deviants would be present only after this point.

^9^It should be noted, however, that this argument is partly impressionistic and based on the experience of language teachers (Riad, 2014).
